# Analysis of Reproduction Number R_0_ of COVID-19 Using Current Health Expenditure as Gross Domestic Product Percentage (CHE/GDP) across Countries

**DOI:** 10.3390/healthcare9101247

**Published:** 2021-09-22

**Authors:** Kayode Oshinubi, Mustapha Rachdi, Jacques Demongeot

**Affiliations:** Laboratory AGEIS EA 7407, Team Tools for e-Gnosis Medical & Labcom CNRS/UGA/OrangeLabs Telecom4Health, Faculty of Medicine, University Grenoble Alpes (UGA), 38700 La Tronche, France; Kayode.Oshinubi@univ-grenoble-alpes.fr (K.O.); Mustapha.Rachdi@univ-grenoble-alpes.fr (M.R.)

**Keywords:** exponential model, COVID-19, ARIMA, current health expenditure as gross domestic product percentage

## Abstract

(1) Background: Impact and severity of coronavirus pandemic on health infrastructure vary across countries. We examine the role percentage health expenditure plays in various countries in terms of their preparedness and see how countries improved their public health policy in the first and second wave of the coronavirus pandemic; (2) Methods: We considered the infectious period during the first and second wave of 195 countries with their current health expenditure as gross domestic product percentage (CHE/GDP). An exponential model was used to calculate the slope of the regression line while the ARIMA model was used to calculate the initial autocorrelation slope and also to forecast new cases for both waves. The relationship between epidemiologic and CHE/GDP data was used for processing ordinary least square multivariate modeling and classifying countries into different groups using PC analysis, K-means and hierarchical clustering; (3) Results: Results show that some countries with high CHE/GDP improved their public health strategy against virus during the second wave of the pandemic; (4) Conclusions: Results revealed that countries who spend more on health infrastructure improved in the tackling of the pandemic in the second wave as they were worst hit in the first wave. This research will help countries to decide on how to increase their CHE/GDP in order to properly tackle other pandemic waves of the present COVID-19 outbreak and future diseases that may occur. We are also opening up a debate on the crucial role socio-economic determinants play during the exponential phase of the pandemic modelling.

## 1. Introduction

Among the main economic indicators used there is the CHE/GDP index, which is the percentage of the gross domestic product (GDP, equal to the total monetary or market value of all the finished goods and services produced within a country’s borders) devoted to the health expenditure by a country (available on the World bank website [1]). This index is high for developed countries (except Japan) and it has been proved that it was correlated to the Gini’s index, which measures the degree of inequality in the distribution of income in a country: to summarize a rich developed or developing country having a large gap between incomes of the richest and poorest parts of the population spends a lot on health both on high-tech care for the rich (usually in the privatized part of the health system) and on essential care for the often unhealthy poor. A poor developing country having a weak Gini’s index spends, more rationally in general, spending for its middle and poor classes. In this article, we will seek to see the relationships between the socio-economic index CHE/GDP and the spread of COVID-19 in countries where the corresponding data are available from the Worldbank [1] and Worldometers [2] websites. 

So far, most countries have experienced at least two peaks of the COVID-19 pandemic and it is necessary to look at both waves and then derive the best conclusion on the efficacy of outlook during these both waves. Health officials, scientists and those involved in the modelling of the pandemic have made a lot of suggestions from the day the first case has been recorded in Wuhan, China. Current health expenditure as gross domestic product percentage (CHE/GDP) is key to different countries’ preparedness to respond for curtailing the pandemic even though it is general belief that no one was prepared during the first wave of the pandemic as most developed nations were worst hit and the death toll increased exponentially.

Our goal is to correlate the maximum basic reproduction number R_0_ of both waves with CHE/GDP. In order to holistically approach this subject, we used many diverse regression tools and also developed some clustering strategies across all countries considered. The results are key in order to protect lives and improve health infrastructure in the future even though we know that the pandemic is still evolving in different countries.

## 2. Materials and Methods 

### 2.1. Materials: The Variables

The variables used for this research are seven in total. The maximum basic reproduction number R_0_ for first and second waves is chosen during the exponential phase of all countries considered. The exponential and autocorrelation slopes are calculated using 100 days from the start of a wave depending on the date a particular country recorded their first case between February and August 2020 while also 100 days was used to calculate for the second wave between 15 October 2020 to 22 January 2021 for all countries considered. The opposite of the initial autocorrelation slope was averaged on six days. CHE/GDP was collated from World Bank data [1]. The deterministic R_0_ was drafted from previous research [3] and it was calculated as the Malthusian growth parameter during the exponential phase of both waves across countries. The daily new cases were drafted from Worldometers^®^ [2] and Renkulab^®^ [4] databases and processed using Python^®^ facilities [5].

### 2.2. Methods

#### 2.2.1. Exponential and ARIMA Model

The exponential model is given as y = a10^bx^, where y is the daily number of new cases, x is the number of days, b is the slope and a is a constant, and the log format can be written as logy = loga + bx. 

ARIMA modelling has been introduced by N. Wiener for prediction and forecasting [5]. Its parametric approach assumes that the underlying stationary stochastic process of the COVID-19 new daily cases N(t) can be described by a small number of parameters using the autoregressive ARIMA model N(t) = Σ_i=1,s_ a(i) N(i) + W(t), where W is a random residual with the aim being to minimize its variance. The autocorrelation analysis is done by calculating the correlation A(k) between the N(t)’s and the N(t − k)’s (t belonging to a moving time window) by using the formula:

(1) $$A\left(k\right)=\frac{E\left[N\left(t\right)-E\left(N\left(t\right)\right)]E[N\left(t-k\right)-E\left(N\left(t-k\right)\right)\right]}{\sigma \left(N\left(t\right)\right)\sigma \left(N\left(t-k\right)\right)}$$

where E denotes the expectation and σ the standard deviation. The autocorrelation function A allows examining the serial dependence of the N(t)’s. We used the ARIMA form of (6, 1, 0), we have shown it was the best for the modelling of the COVID-19 outbreak [6].

#### 2.2.2. Clustering Methodology

Clustering is a branch of machine learning which is called ‘unsupervised learning’ and is frequently utilized to classify biomedical data. We used three classical clustering methods, K-means, PCA (principal component analysis) and hierarchical clustering [6]. K-means clustering chooses a priori the number of clusters and starts out with random centroids while hierarchical clustering starts with every point in dataset as a cluster, then finds the two closest points and combines them into clusters, the process being repeated until appears a big giant cluster and it then creates a dendrogram. 

Principal component analysis (PCA) also helps to cluster data points and it is also one of dimension reduction techniques because each variable has a different dimension. It allows us to summarize and visualize the information in a data set described by multiple inter-correlated variables. PCA is used to extract the important information from variables in the dataset and to express this information as a set of few new variables called principal components (PC’s).

#### 2.2.3. Linear and Polynomial Regression

Linear regression models use some historic data (100 days infectivity period in our case) of independent and dependent variables (CHE/GDP) and consider a linear relationship between both while polynomial regression models use a similar approach but the dependent variable is modeled as a degree *n* (6 ≥ *n* ≥ 2) polynomial in x.

#### 2.2.4. Multivariate Ordinary Least Square Method

Multivariate least squares method allows us to test much more complex relations between variables. It can be can be represented as follows:

(2) $$y=\beta_{1}x_{1}+\beta_{2}x_{2\;}+\cdots +\in ,\;$$

where $\beta_{1},\beta_{2\;},\cdots$ are coefficients or weights, ∈ is the residual noise, y is the dependent variable and $x_{1},x_{2\;},\cdots$ are the independent variables.

## 3. Results

### 3.1. Autocorrelation Slope

#### 3.1.1. Parabolic and Cubic Regression

**Figure 1:**
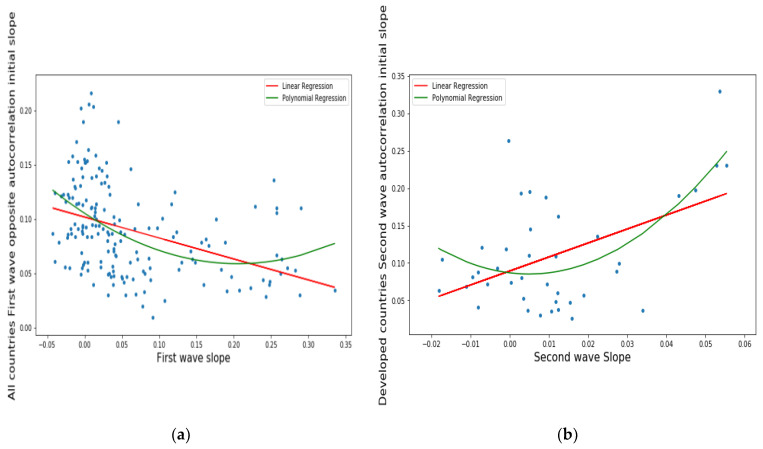

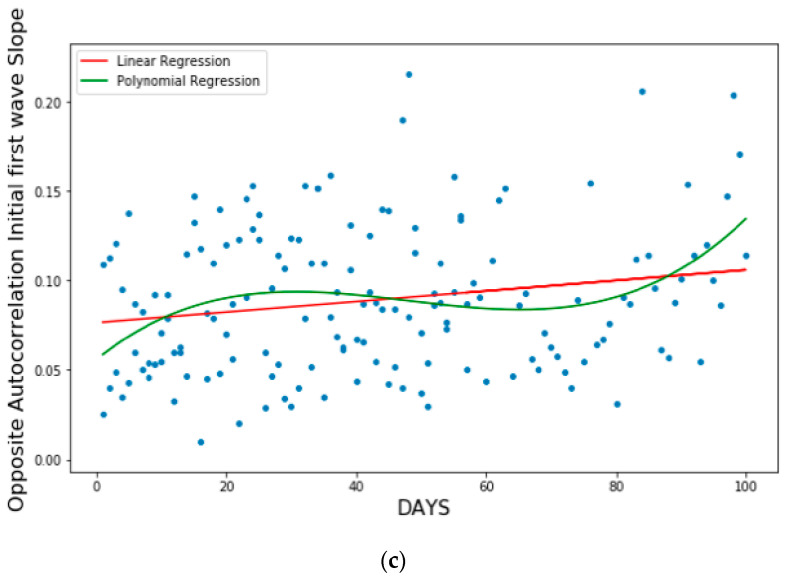
Linear (in red) and parabolic or cubic (in green) regression plots of the opposite of the initial autocorrelation slope vs. (**a**) first wave exponential regression slope for all countries, (**b**) second wave exponential regression slope for developed countries and (**c**) days from the start of the first wave observed in China for all countries. (**a**): LinregressResult slope = −0.193, intercept = 0.102, *r* value = −0.394, *p* value = 1.026 × 10^−7^, stderr = 0.03467, *p* value = 0.00145, stderr = 0.54339, R-squared for order two polynomial regression = 0.19, RMSE for linear regression = 0.0385, RMSE for polynomial regression = 0.046, (**b**): LinregressResult slope = 1.867, intercept = 0.089, *r* value = 0.487, R-squared for order two polynomial regression = 0.37, RMSE for linear regression = 0.063, RMSE for polynomial regression = 0.094, (**c**): LinregressResult slope = 0.000295, intercept = 0.0765, *r* value = 0.195469, *p* value = 0.01415, stderr = 0.000119, R-squared linear regression = 0.038, R-squared for order three polynomial pegression = 0.1, RMSE for linear regression = 0.04, RMSE for polynomial regression = 0.0414825.

The meaning of the abbreviations used in Figure 1 is the following: LinregressResult slope = slope of the linear regression, intercept = ordinate at origin of the regression curve, *r* value = correlation coefficient, *p* value = *p* value of the nullity test of correlation coefficient, stderr = standard error of the regression, RMSE = root of mean square error.

Figure 1 aims to show that classical linear and polynomial regressions (parabolic for Graphs (a) and (b) and cubic for Graph (c)) between the opposite of the slope at the origin of the autocorrelation function of the ARIMA model and successively the slope of the logarithmic regression line of the new daily cases of COVID-19 of the first wave (a), then that of the second wave (b), and finally the number of days since the start of the outbreak (c). 

The curves show a different behavior between the two waves (a) and (b), probably due to an increase in the contagion parameter, the basic reproduction number R_0_ (linked to the Malthusian parameter of the exponential growth phase), despite a shortening of the duration of contagiousness (linked to the slope at the origin of the autocorrelation function, which is all the stronger as the distance from the start of the epidemic increases, no doubt because of the mitigation measures, which decrease the duration of the contagiousness period).

#### 3.1.2. Quartic Regression

**Figure 2:**
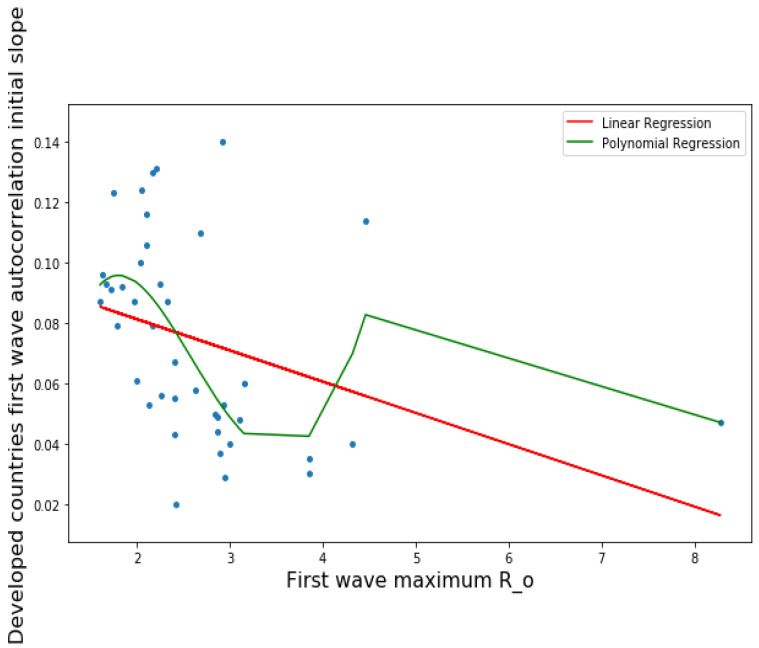
Linear (in red) and quartic (in green) regression plots of the opposite of the initial autocorrelation slope of the first wave vs first wave maximum R_0_ for developed countries. LinregressResult slope = 0.01034, intercept = 0.1019, *r* value = −0.3578, *p* value = 0.02163, stderr = 0.00433, RMSE for linear regression = 0.0303, RMSE for polynomial regression = 0.0349, R-squared for order four polynomial regression = 0.33.

We have used in Figure 2, a polynomial of degree 4 for obtaining a fit showing a minimum for the value of the maximum R_0_ equal to 3.5, which is considered as the observed value for the maximal effective reproduction number at start of the first wave in many developed countries (France, Germany, Switzerland, UK, USA, etc.) [4], which corresponds to the fact that the opposite of the initial autocorrelation slope (indicating that the length of the contagiousness is short when the absolute value of the slope is high) decreases (the contagiousness duration increases) when the maximum R_0_ increases, which seems logical.

#### 3.1.3. Sextic Regression

**Figure 3:**
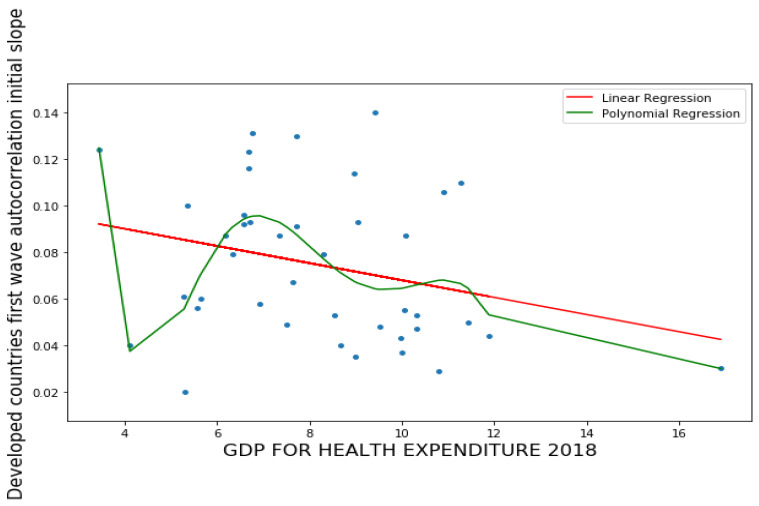
Linear (in red) and sextic (in green) regression plots of first wave opposite of initial autocorrelation slope vs. CHE/GDP. LinregressResult slope = 0.01117, intercept = 0.0664, *r* value = 0.47219, *p* value = 0.0097, stderr = 0.004, RMSE for linear regression = 0.0387, RMSE for polynomial regression = 0.04399, R-squared for order six polynomial regression = 0.4.

We studied the correlation between the value of the opposite of the slope at the origin of the autocorrelation function of the first wave and the economic and health index CHE/GDP, by studying a polynomial regression of degree 6 (Figure 3). It shows an anticorrelation in the linear regression and a local maximum for countries with an average CHE/GDP ratio of around 7. Countries with a high CHE/GDP ratio (such as France and the United States) have a low value of l in opposite to this slope. The explanation for this phenomenon may come from the correlation reported in the introduction between the CHE/GDP and Gini indices, the poor classes having a longer duration of contagiousness due to a less important state of immunological defense and perhaps less compliance with mitigation measures.

### 3.2. Exponential Model Slope

#### 3.2.1. Developed and Developing Countries

**Figure 4:**
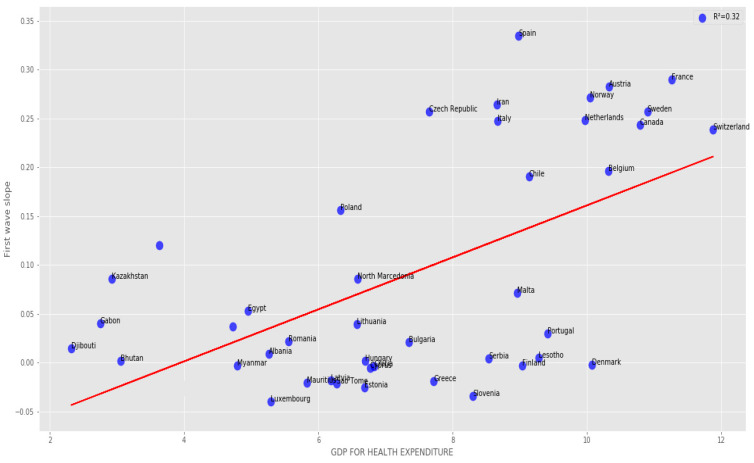
Regression plot of first wave exponential regression slope vs CHE/GDP for developed and developing countries. LinregressResult slope = 0.026632, intercept = −0.1052912, *r* value = 0.5661, *p* value = 7.60 × 10^−5^, stderr = 0.00605655, R-squared = 0.320470, RMSE for linear regression = 0.095836.

**Figure 5:**
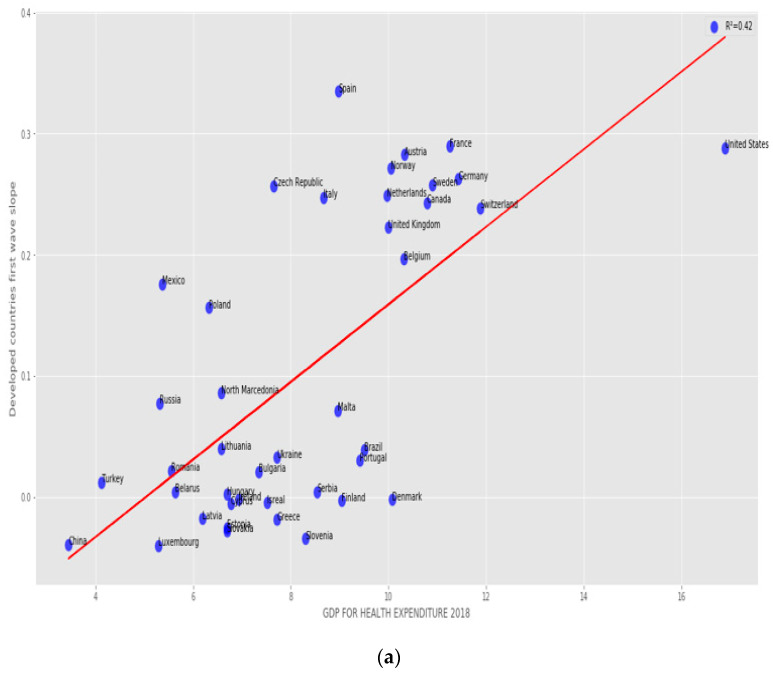

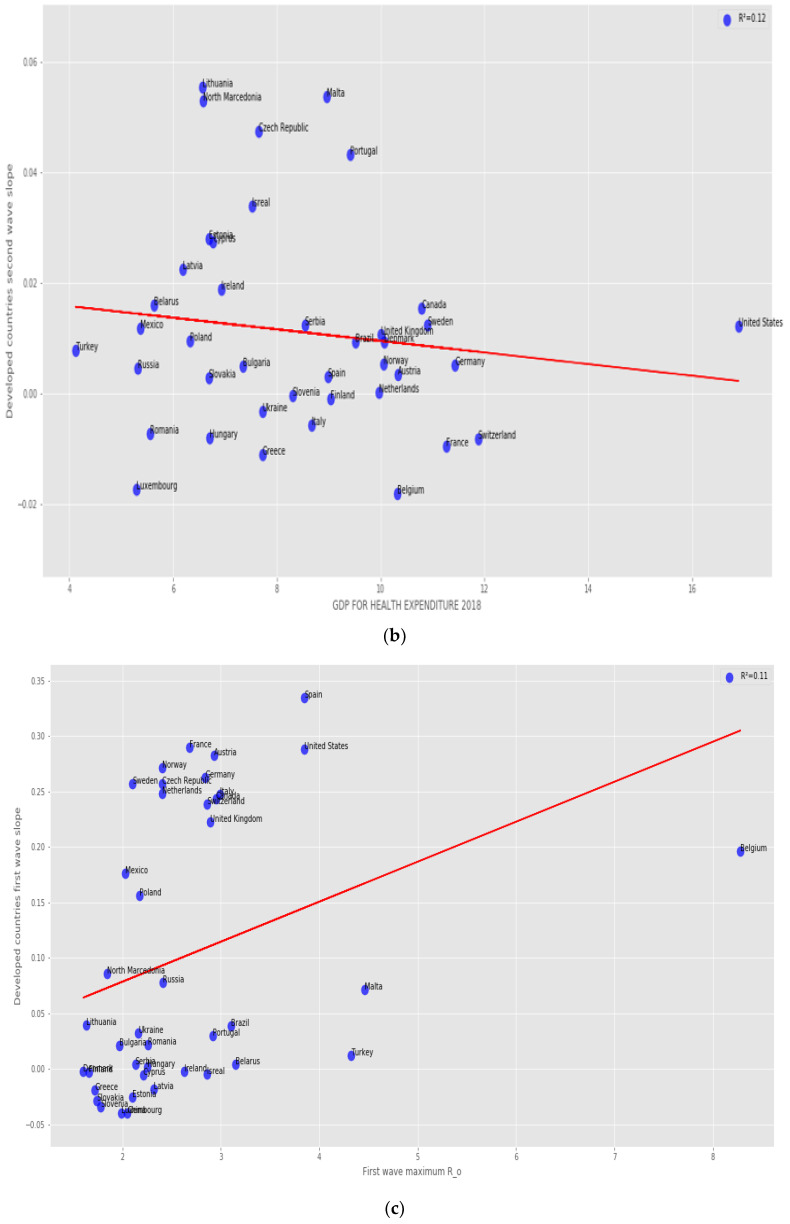

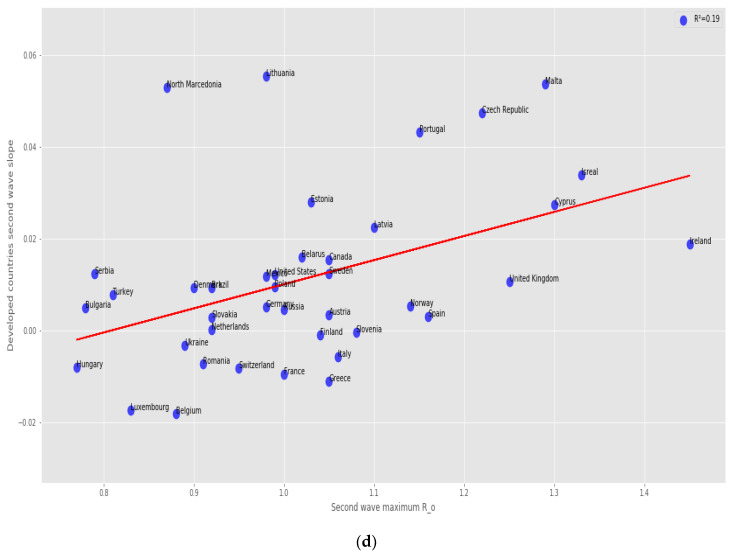
Regression plots for developed countries of (**a**) first and (**b**) second wave exponential regression slope versus CHR/GDP, (**c**) first and (**d**) second wave maximum R_0_ of the new cases curve. (**a**): LinregressResult slope = 0.0320468, intercept = −0.16158, *r* value=0.6481, *p* value = 4.62 13 × 10^−6^, stderr = 0.00603, R-squared = 0.42, RMSE for linear regression = 0.09359760581, (**b**): LinregressResult slope = −0.0010489, intercept = 0.01994, *r* value = 0.1340845, *p* value = 0.4094462, stderr = 0.001258, R-squared = 0.018, RMSE for linear regression = 0.018583749, (**c**): LinregressResult slope = 0.03612, intercept = 0.0062, *r* value = 0.3299, *p* value = 0.0352, stderr = 0.0165, R-squared = 0.109, RMSE for linear regression = 0.116, (**d**): LinregressResult slope = 0.05223, intercept = −0.0421, *r* value = 0.434366, *p* value = 0.0051, stderr = 0.01757, R-squared = 0.18867, RMSE for linear regression = 0.01689.

The correlation between the first wave exponential regression slope and the CHE/GDP index for developed and developing countries is significantly positive (R = 0.57) on Figure 4. Figure 5 shows the same but for developed countries only with a still higher correlation (R = 0.65).

#### 3.2.2. Developed Countries

The correlation between the first wave exponential regression slope and the CHE/GDP index for developed and developing countries is significantly positive (R = 0.57) on Figure 4. Figure 5 shows the same but for developed countries only with a higher correlation (R = 0.65).

#### 3.2.3. All Countries

**Figure 6:**
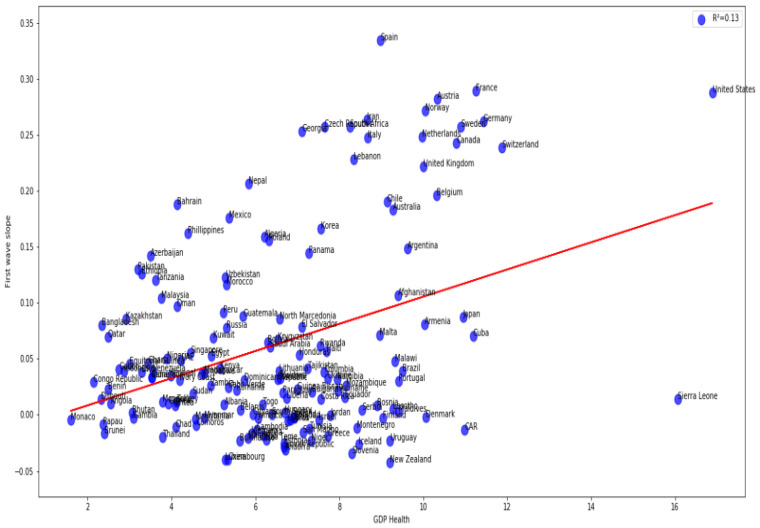
Regression plot of first wave exponential regression slope vs CHE/GDP for all countries. LinregressResult slope = 0.01214439, intercept = −0.0159087, *r* value = 0.3655, *p* value = 2.71 × 10^−6^, stderr = 0.00249223, R-squared = 0.13359, RMSE for linear regression = 0.0819603345.

Figure 4 and Figure 5 show a positive correlation between the slope of the logarithmic regression curve of the new cases of COVID-19 as a function of time (a sign of rapid growth of the epidemic if it is high) and the economic index CHE/GDP. This is true when we observe the developed and developing countries (Figure 4) or the developed countries alone for which the positive correlation is higher, the correlation coefficient de correlation increasing from 0.57 to 0.65 (Figure 5a), but this trend is reversed for the second wave (Figure 5b), where the correlation coefficient equal −0.57, this being possibly due the early implementation of mitigation measures in developed countries, reducing the exponential growth of new cases in the second wave. This trend is confirmed in the study of the correlation between the slope of the logarithmic regression and the maximum R_0_ (Figure 5c,d), which increases during the second wave in developed countries (the correlation coefficient rising from 0.33 to 0.44), showing a growth of the new cases more brutal, but shorter, undoubtedly due to the establishment of a faster and more effective lockdown. This correlation coefficient for the first wave remains for all countries close to that for developed countries (Figure 6).

### 3.3. ARIMA Model for First and Second Wave

**Figure 7:**
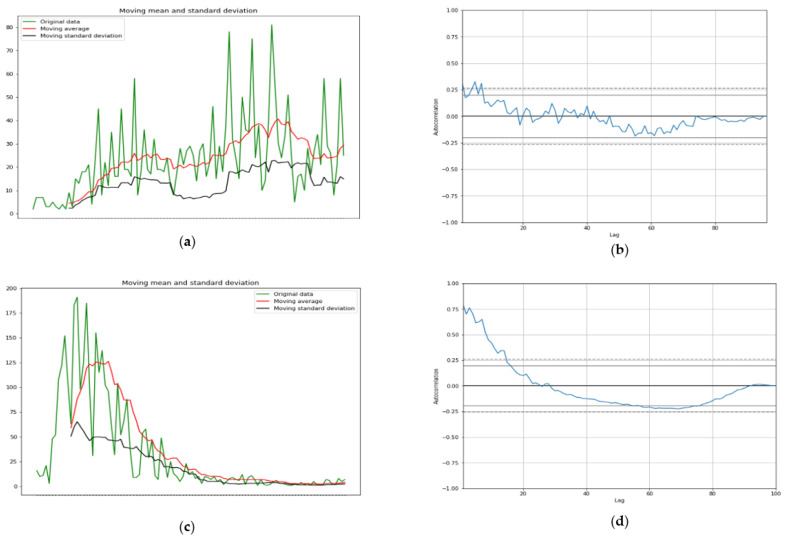
(**a**) First wave moving average and standard deviation of new cases (left) and (**b**) autocorrelation curve for Mali (right). (**c**) First wave moving average and standard deviation of new cases (left) and (**d**) autocorrelation curve for Luxembourg (right).

The ARIMA model shows more than 95% confidence interval as it can be seen in Figure 7a–d with p value for Mali for first wave is *p* = 0.01 and for second wave it is *p* = 6.3 $\times 10^{-10}$ while for first wave for Slovenia *p* = 0.01 and for second wave in Luxembourg *p* = 0.01. 

#### 3.3.1. First Wave ARIMA Model

**Figure 8:**
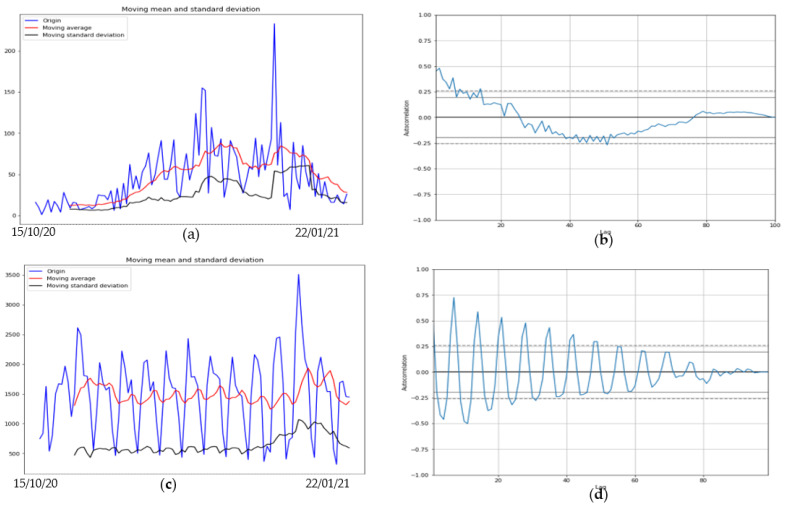
(**a**) Second wave moving average and standard deviation of new cases (left) and (**b**) autocorrelation curve for Mali (right). (**c**) Second wave moving average and standard deviation of new cases (left) and (**d**) autocorrelation curve for Slovenia (right).

The comparison during the first wave between two countries (Figure 7), one developed (Luxembourg) and one developing (Mali) shows a difference in length of contagiousness period (linked to the value of the opposite to the slope at origin of the autocorrelation function) and shape of the growth curve, indicating a lower virulence of the SARS Cov-2 in Mali, possibly due to the influence of the temperature [7]. This tendency is reversed during the second wave between Mali and Slovenia (Figure 8).

#### 3.3.2. ARIMA Model Forecast for First and Second Wave

**Figure 9:**
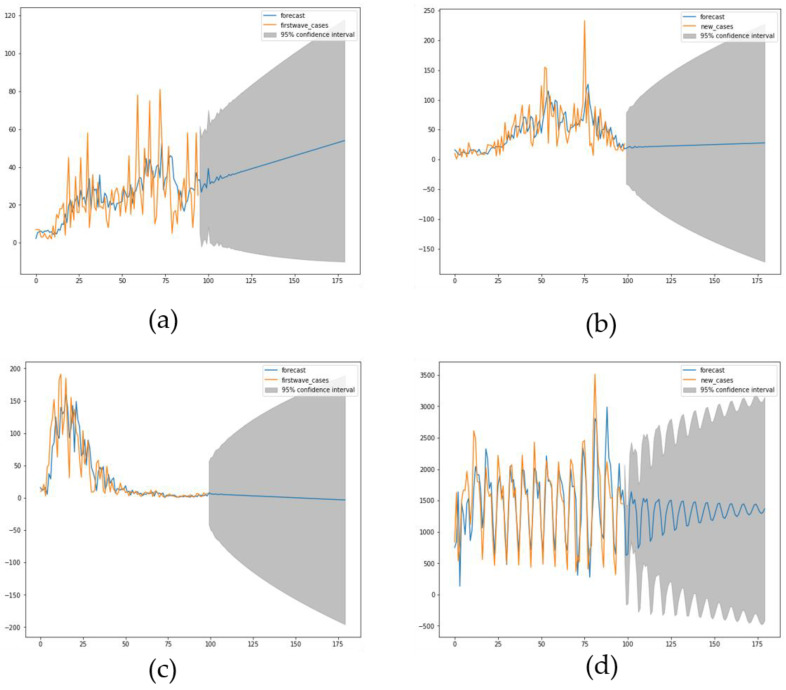
(**a**) First and (**b**) second wave forecast for Mali. (**c**) First wave forecast for Luxembourg. (**d**) Second wave forecast for Slovenia.

The forecast using the ARIMA method shows a good retrospective adjustment to past data, but a weak predictive power of the future trend of new cases, in particular for the prediction of the entry into the endemic phase after an epidemic wave (Figure 9).

### 3.4. Clustering of Countries from Epidemic and Economic Variables

**Figure 10:**
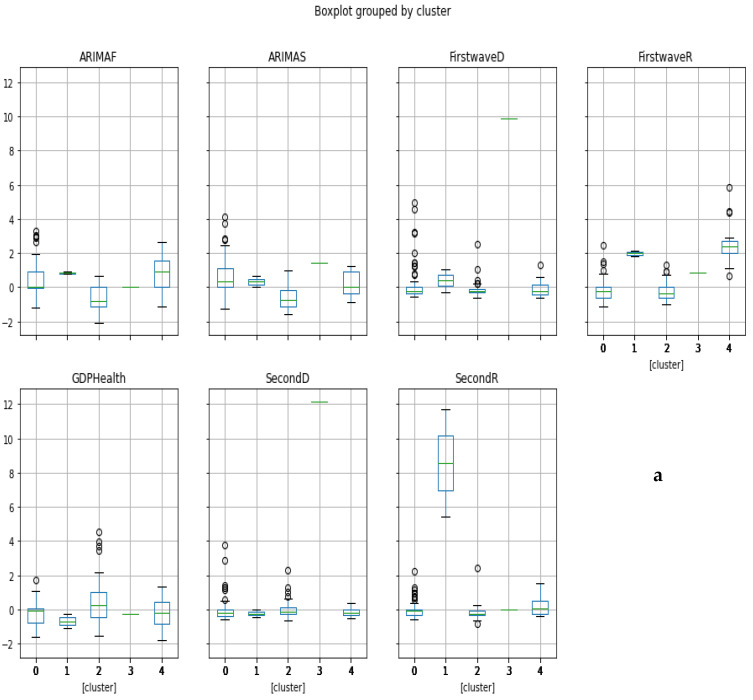

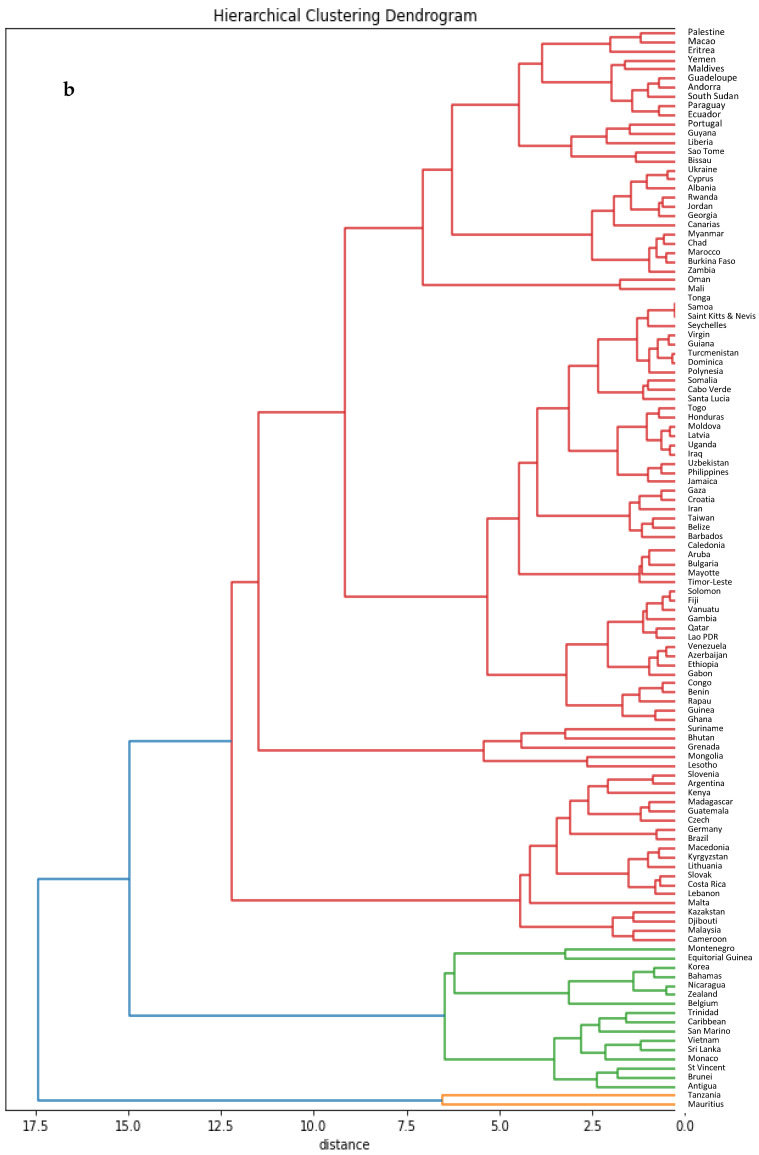

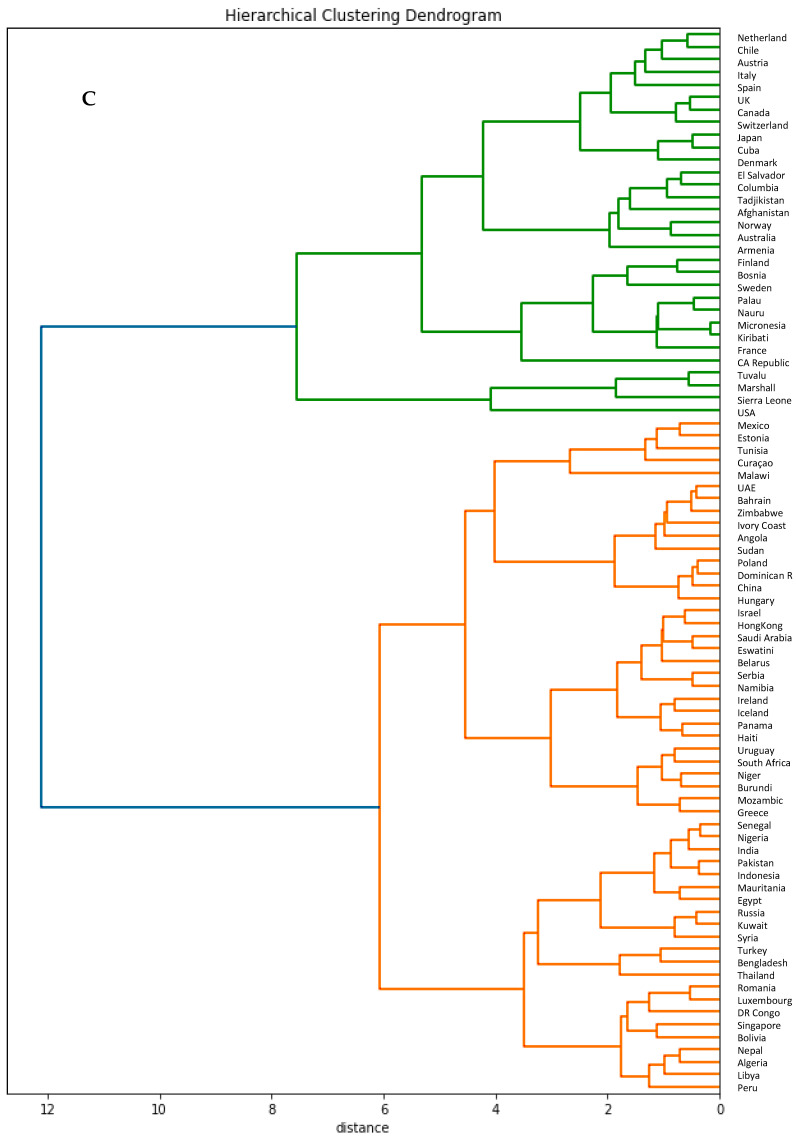
(**a**) Boxplots of the clusters corresponding to the hierarchical clustering. Visualizations of (**b**) more “developing” (in red with some notable exceptions such as the Czech Republic and Germany) and (**c**) more “developed” (in green and partially in orange) countries parts of the hierarchy tree.

**Figure 11:**
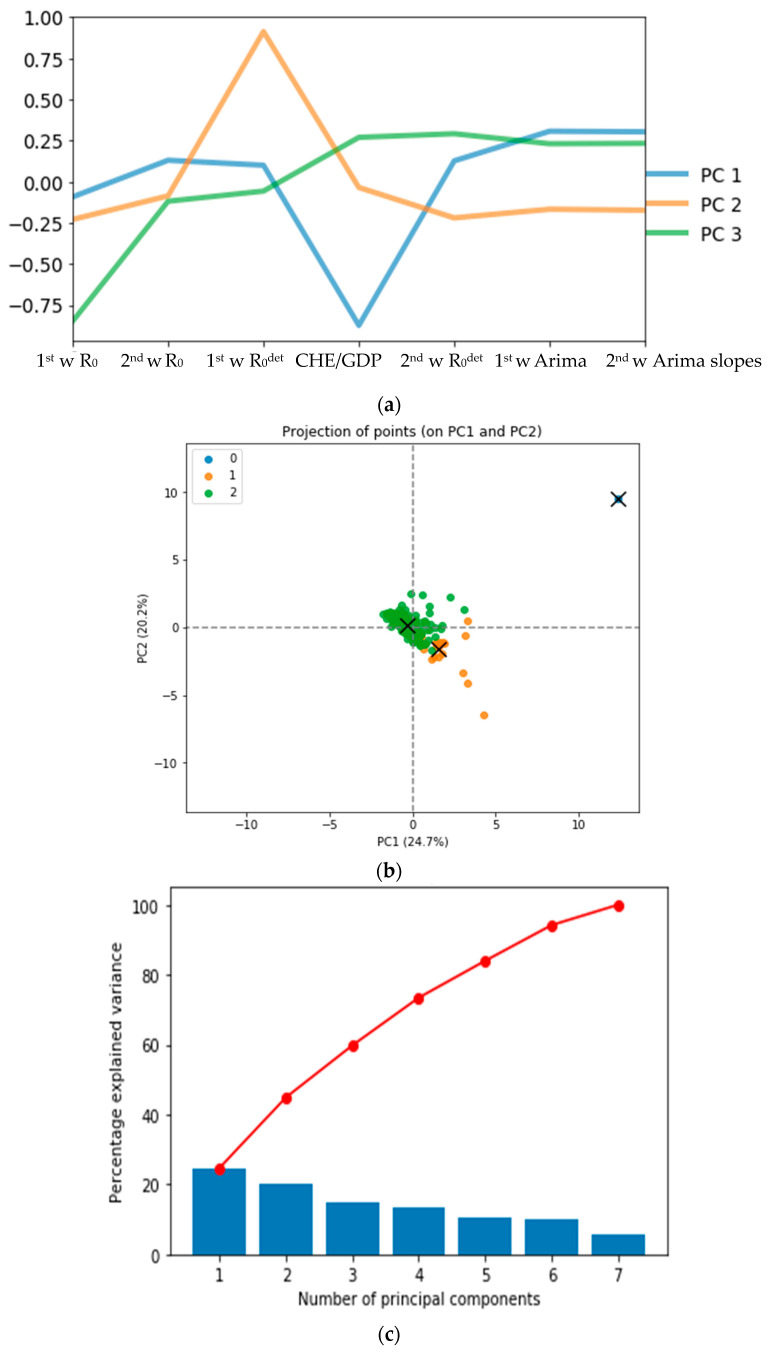

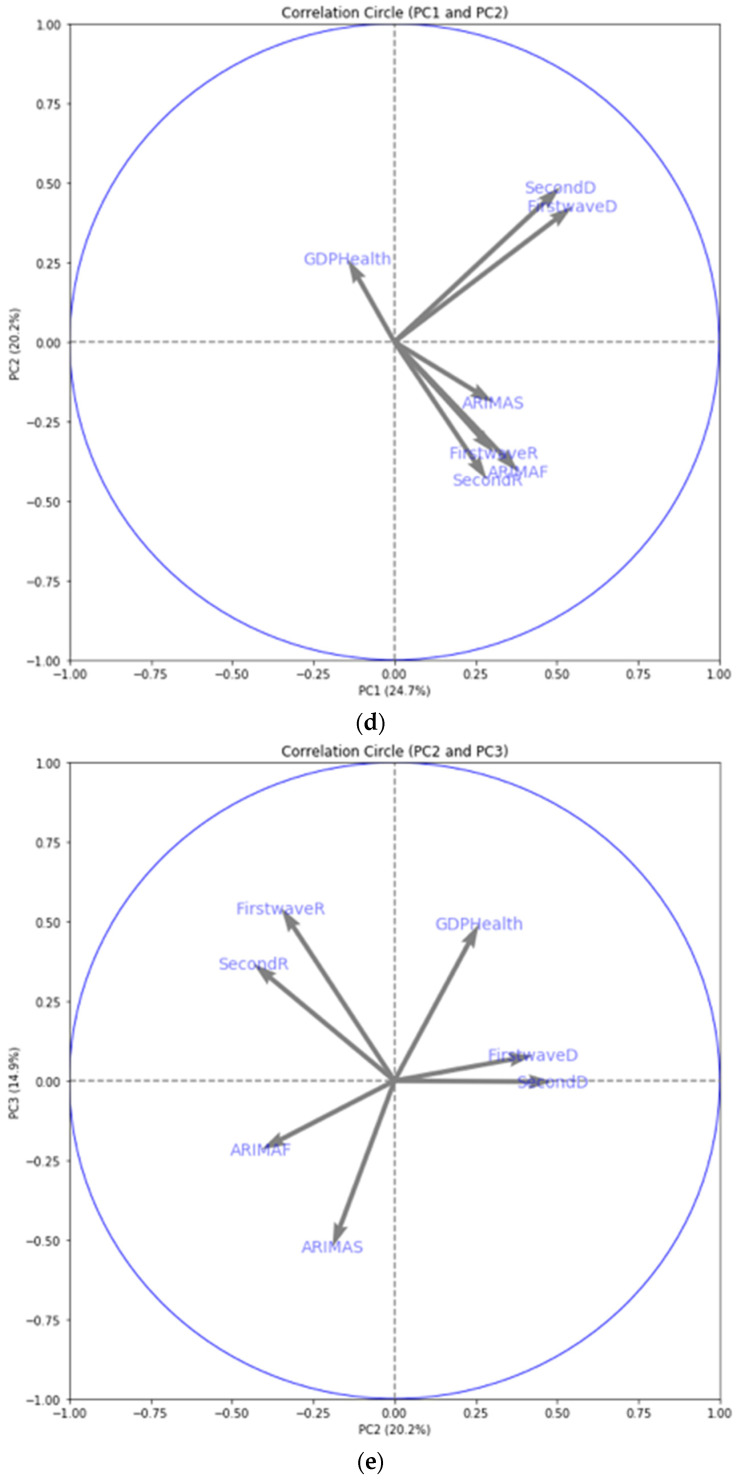
(**a**) Principal components (PC) plot from the principal component analysis (PCA) on the initial variables: 1st and 2nd waves maximum R_0_, first wave R_0_ and second wave R_0_, deterministic R_0_, 1st wR_0_^det^ and 2nd wR_0_^det^, 1st wave Arima slope, 2nd wave Arima slope, and CHE/GDP. (**b**) Projection of the points corresponding to 204 countries of the PCA’s plot on the first PC plane with more developed countries in green and more developing in orange. (**c**) Explained variance plot. (**d**,**e**) Correlation circles for the two first PC planes.

**Figure 12:**
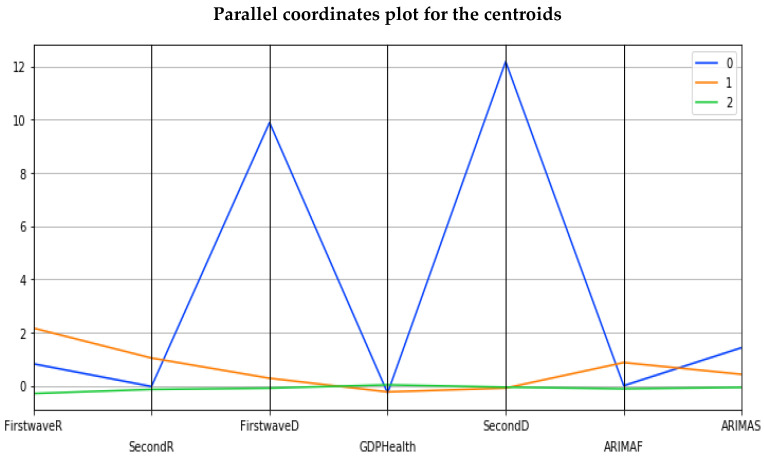
Parallel coordinates for cluster centroids.

The hierarchical clustering allows developed and developing countries to be grouped into 5 separate clusters (Figure 10) and Principal Component Analysis into 3 separate clusters (Figure 11), one being a singleton corresponding to Spain (Figure 12). 

### 3.5. Ordinary Least Square Method. The Multivariate Case

**Figure 13:**
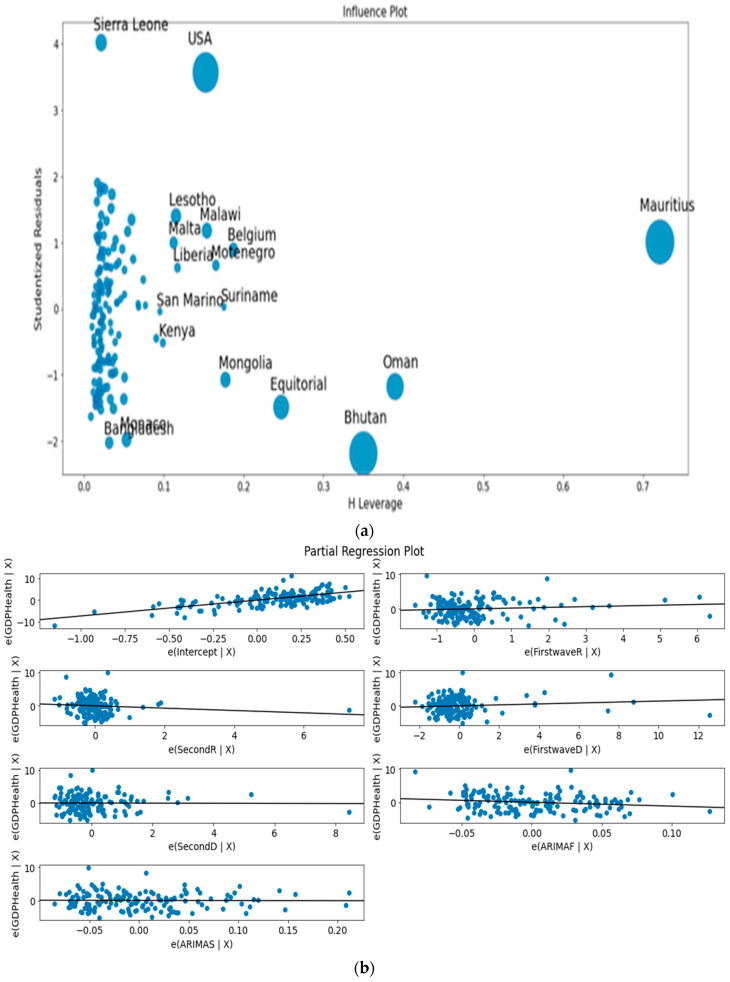
(**a**) Leverage vs normalized squared residuals plot. (**b**) Residuals regression plots for initial variables.

The clustering of the countries from epidemic and economic variables is described in Figure 10, Figure 11, Figure 12 and Figure 13 and shows several features:

1. The hierarchical clustering (Figure 10 and Figure 11b) shows a trend common to developed countries (shown in green), with the notable exception of Germany and Czechia,
2. The principal component analysis shows the importance of the CHE/GDP index in the first principal component (Figure 11a,c,d) and of the deterministic R_0_ (R_0_^det^) of the exponential phase of the first wave in the second principal component and of the second wave in third principal component (Figure 11e);
3. The analysis of parallel coordinates for cluster centroids also shows the importance of the deterministic R_0_ in the discrimination of clusters (Figure 12);
4. The analysis of the residuals shows a good explanatory power of the first three principal components (60% of the total variance in Figure 11c, confirmed by the projections on the two first principal planes of Figure 11d,e), and a weak correlation of the principal components with these residuals (Figure 13a,b).

## 4. Discussion

There are a lot of differences between the first and second wave results concerning the exponential regression slope and the autocorrelation initial slope: while some countries have higher figures for the first wave, others have lower figures for the second wave and vice versa. This was also evident for the regression plot where some countries have negative correlation values for the first wave of some growth parameters with the CHE/GDP and positive for the second wave, and vice versa for other countries. These phenomena prove that the way the pandemic spread in the second wave is different from what was experienced in the first wave. In the principal component analysis, we discovered that first wave deterministic R_0_ and CHE/GDP health had high weights in first and second principal components (PC1 and PC2), which are dominant components in the PC analysis. 

More precisely, on Figure 1a,b first and second waves of the COVID-19 pandemic are compared using linear and parabolic or cubic regression, showing a significant positive (resp. negative) correlation between the opposite of the initial autocorrelation slope and exponential regression slope of the first (resp. second) wave for developed (resp. all) countries. This opposition between the two waves could result from the application of a more severe lockdown in developed countries during the second wave. On Figure 1c, the opposite of the initial autocorrelation slope decreases significantly if the start of the first wave in a country is late with respect to the start of the COVID-19 outbreak in China due probably to the progressive implementation of mitigation measures in that country taking into account the experience of the countries starting first wave before. On Figure 2, the opposite of the initial autocorrelation slope is significantly negatively correlated with the maximum R_0_ observed at the inflection point of the new cases curve, confirming that long contagiousness periods give high exponential increases of the new cases. On Figure 3, for the first wave the opposite of the initial autocorrelation slope is positively (resp. negatively) correlated with the CHE/GDP (resp. maximum R_0_) for developed countries, which could correspond to the efficiency of the mitigation measures decided in these countries. This is confirmed on Figure 4, where the first wave exponential regression slope is positively correlated with the CHE/GDP in a mix of developed and developing countries. The Figure 5a shows the same type of effect of public health policies in developed countries for the first wave, where CHE/GDP increases with the first wave exponential regression slope, but this result is inverted on Figure 5b for the second wave perhaps due to a rationalization of the care activity between the first two waves. Figure 5c,d shows a similar behavior of the two waves concerning the positive correlation between the exponential regression slope and the maximum R_0_, which makes sense, as these quantities are both related to the initial exponential growth of an epidemic wave. For the first wave of all countries, Figure 6 shows the same positive correlation as Figure 5a between the exponential regression slope and CHE/GDP.

Figure 7 compares two countries, one from Sahelian Africa, Mali and one from western Europe, Luxembourg during the first wave of Covid-19 outbreak during the spring 2020: Mali shows a quasi-endemic behavior with a weakly varying autocorrelation function and Luxembourg a frank epidemic wave with a classic shape. For the second wave in fall 2020, Mali presents an attenuated epidemic shape (due probably to specific geoclimatic conditions in western Africa [7]) and a country from central Europe, Slovenia, shows at this period an endemic behavior with an oscillatory occurrence of new cases. Figure 9 proposes a forecasting based on ARIMA decomposition for the first and second waves in Mali with a better approximation for the epidemic second wave than for the quasi-endemic first wave. It is the same for Luxembourg with an inversion of the phases order, an epidemic wave followed by an endemic state well predicted. On the contrary, for Slovenia, the endemic state with oscillations is badly predicted.

Clustering of all countries is then studied on Figure 10, Figure 11 and Figure 12. Figure 10a shows the boxplot of the seven initial variables used in hierarchical clustering: the first and second wave opposite of the initial autocorrelation slope (respectively ARIMAF and ARIMAS), exponential regression slope and maximum R_0_ (respectively FirstwaveD, SecondD, FirstwaveR, SecondR), and CHE/GDP. The boxplots contain five clusters represented in Figure 10b,c corresponding to more “developing” (in red with some notable exceptions such as the Czech Republic and Germany) and (c) more “developed” (in green and partially in orange) countries parts of the hierarchy tree, with a small “exotic” cluster for Tanzania and Mauritius. Figure 11a–e shows the results of the principal component analysis (PCA), with (a) the three principal components declined on the initial variables calculated for all countries (first and second waves maximum R_0_’s denoted first wR_0_ and second wR_0_, deterministic R_0_’s denoted first wR_0_^det^ and second wR_0_^det^, Arima slopes denoted first wArima, second wArima slopes, and the current health expenditure as gross domestic product percentage denoted CHE/GDP), (b) the projection of the points corresponding to countries of the PCA’s plot on the first PC plane, (c) the explained variance plot and (d,e) the correlation circles for the first three principal components with projection of the initial variables as vectors (having 195 components corresponding to the 195 countries of the Table A1 in Appendix A) on the corresponding principal planes. In Figure 11a, the main initial variable in the linear combination of the first (resp. the second) principal component is the first wave deterministic R_0_^det^ (resp. the CHE/GDP) and these two initial variables R_0_^det^ and CHE/GDP are anticorrelated as we have already noticed when commenting before on the Figure 3 (a country devoting a large share of its GDP to health expenditure reduces the occurrence of new cases). Figure 11b gives the projection of 204 countries on the first PC plane and distinguishes two main clusters of 118 and 85 countries, respectively, plus a singleton representing Botswana, with more developed countries in green and more developing countries in orange. Figure 11c shows that 60% of the variance is explained by the three first PCs, and Figure 11d,e presents the correlation circles with projection of the initial variables as vectors on the corresponding two principal planes (PC1, PC2) and (PC2, PC3), showing such as in Figure 11a the preeminence of the opposite vectors, the first wave deterministic R_0_ and the CHE/GDP. Figure 12 shows also for the first k-means cluster the importance of the first wave deterministic R_0_. 

Finally, Figure 13 a,b corresponds to the ordinary multivariate least square method. Figure 13a shows the eccentric position of developed countries such as Belgium and USA and developing countries such as Equatorial Guinea and Suriname as outliers not fitting the data bulk, and Figure 13b the concentration of the initial variable CHE/GDP with the first and second waves deterministic R_0_^det^, in agreement with the fact that they are the most dominant initial variables in PCA and k-means clustering.

## 5. Conclusions

We have shown in this article that there exist correlations between the growth parameters directly linked to the occurrence of new cases of COVID-19 and socio-economic variables, in particular the current health expenditure as gross domestic product percentage (CHE/GDP) anticorrelated with the basic reproduction time R_0_, which shows the effectiveness of public health mitigation measures, even if they involve significant medico-economic costs. Larger perspectives are offered by combining this study with others on geoclimatic and demographic severity factors of the COVID-19 outbreak [7,8] with the present socio-economic determinants, in order to obtain the most comprehensive and accurate picture of non-biological exogenous influences on the expanding COVID-19 pandemic. 

Concerning the contagious diseases, public health physicians and policy-makers are constantly faced with four challenges. The first concerns the estimation of the basic reproduction number R_0_. The systematic use of R_0_ simplifies the decision-making process by policy-makers, advised by public health authorities, but it is too caricature to account for the biology behind the viral spread. We have observed that R_0_ was not constant during an epidemic wave due to exogenous and endogenous factors influencing both the duration of the contagiousness period and the transmission rate during this phase. Then, the first challenge concerns the estimation of the mean duration of the contagiousness period for infected patients. As for the transmission rate, realistic assumptions made it possible to obtain an upper limit to this duration [9,10,11], in order to better guide the individual quarantine or lockdown measures decided by the authorities in charge of public health. This upper bound also makes it possible to obtain a lower bound for the percentage of unreported infected patients, which gives an idea of the quality of the census of cases of infected patients, which is the second challenge facing specialists of contagious diseases. The third challenge is the estimation of the daily reproduction numbers over the contagiousness period [2] and the fourth and final interesting challenge is the extension of the methods developed in the present paper to contagious non-infectious diseases (i.e., those without causal infectious agents), such as social contagious diseases, the best example being that of the pandemic linked to obesity, for which many concepts and modelling methods presented here remain available. 

## Data Availability

Data comes from public databases accessible online.

## Abbreviations

ARIMAF	Opposite autocorrelation slope for first wave
ARIMAS	Opposite autocorrelation slope for second wave
SecondR	Maximum R_0_ for second wave from [3]
SecondD	Deterministic R_0_ for second wave from [2]
FirstwaveR	Maximum R_0_ for first wave from [3]
FirstwaveD	Deterministic R_0_ for first wave from [2]
CHE/GDP	Current health expenditure as gross domestic product percentage [1]

## Appendix A

**Table A1:** Parameters for first and second waves for 195 countries.

All Countries		First Wave				Second Wave				
No.	Country Name	$R_{o}$	$D-R_{o}$	Exponential Slope	Auto-Correlation Slope	$R_{o}$	$D-R_{o}$	Exponential Slope	Auto-Correlation Slope	GDP Health 2018
1	AFGHANISTAN	1.78	0.65	0.1070	−0.025	0.79	0.04	0.0017	−0.097	9.40
2	ALGERIA	2.19	1.25	0.1594	−0.040	0.86	0.91	0.0316	−0.100	6.22
3	ARUBA	-	5.46	-	-	-	1.10	0.0172	−0.112	-
4	ANDORRA	-	1.36	−0.0313	−0.121	-	0.12	−0.0067	−0.155	6.71
5	ANGOLA	2.06	0.63	0.0100	−0.095	1.13	1.70	−0.0135	−0.057	2.55
6	ANTIGUA	4.23	1.92	-	-	3.30	2.13	0.0051	−0.177	5.23
7	ALBANIA	1.61	0.96	0.0091	−0.138	0.99	0.66	0.0058	−0.085	5.26
8	ARGENTINA	2.06	0.73	0.1485	−0.060	1.19	0.36	0.0427	−0.240	9.62
9	ARMENIA	1.51	4.43	0.0809	−0.050	0.80	0.86	0.0570	−0.090	10.03
10	AUSTRALIA	2.45	2.79	0.1832	−0.054	1.11	1.50	0.0037	−0.136	9.28
11	AUSTRIA	2.93	1.17	0.2825	−0.053	1.05	2.08	0.0034	−0.053	10.33
12	AZERBAIJAN	2.11	1.16	0.1422	−0.071	0.63	0.37	0.0676	−0.130	3.51
13	BAHAMAS	6.33	0.57	-	-	1.48	1.22	−0.0250	−0.077	6.25
14	BAHRAIN	1.81	1.10	0.1884	−0.079	1.24	1.14	0.0012	−0.053	4.13
15	BANGLADESH	3.67	1.04	0.0799	−0.033	0.92	0.99	−0.0086	−0.046	2.34
16	BARBADOS	4.63	1.86	-	-	1.99	1.14	0.0378	−0.109	6.56
17	BELARUS	3.15	1.57	0.0043	−0.060	1.02	1.07	0.0159	−0.026	5.64
18	BELGIUM	8.28	0.43	0.1963	−0.047	0.88	2.23	−0.0182	−0.063	10.32
19	BELIZE	3.74	0.99	-	-	1.34	0.51	−0.0004	−0.140	5.69
20	BENIN	2.16	0.85	0.0226	−0.133	1.55	0.85	0.0020	−0.125	2.49
21	BHUTAN	2.10	15.00	0.0021	−0.118	2.49	1.08	0.0126	−0.099	3.06
22	BOLIVIA	1.46	2.17	0.0647	−0.045	1.45	1.61	0.0152	−0.087	6.30
23	BOSNIA	1.70	0.09	0.0088	−0.110	0.97	1.56	−0.0118	−0.106	8.90
24	BOTSWANA	3.76	28.47	-	-	1.43	28.43	0.0030	−0.186	5.85
25	BRAZIL	3.10	0.77	0.0389	−0.048	0.92	0.46	0.0092	−0.188	9.51
26	BRUNEI	5.00	1.08	−0.0165	−0.120	3.66	1.00	-	-	2.41
27	BULGARIA	1.97	5.06	0.0178	−0.087	0.78	0.75	0.0049	−0.110	7.35
28	BURKINA FASO	2.44	1.08	−0.0227	−0.123	1.18	0.94	0.0360	−0.058	5.63
29	BURUNDI	2.80	1.33	-	-	1.69	2.18	0.0226	−0.063	7.74
30	CABO VERDE	1.54	0.82	0.0247	−0.091	1.71	0.19	−0.0064	−0.110	5.36
31	CAMBODIA	5.55	0.34	−0.0129	−0.129	3.12	0.27	0.0010	−0.158	6.03
32	CAMEROON	2.56	2.17	0.0338	−0.123	1.64	2.48	0.0085	−0.207	-
33	CANADA	2.95	1.10	0.2432	−0.029	1.05	0.44	0.0153	−0.047	10.79
34	CAR	2.45	1.66	−0.0130	−0.096	4.99	0.33	-	-	10.99
35	CHAD	2.43	1.19	−0.0108	−0.114	1.44	0.77	0.0222	−0.050	4.10
36	CHILE	2.42	1.00	0.1906	−0.034	1.16	1.64	0.0586	−0.090	9.14
37	CHINA	2.05	1.10	−0.0602	−0.088	1.07	0.87	0.0137	−0.068	5.35
38	COLUMBIA	1.86	1.00	0.0384	−0.040	0.99	1.47	0.0061	−0.126	7.64
39	COMOROS	1.93	3.75	−0.0094	−0.153	1.58	1.65	0.0397	−0.076	4.59
40	CONGO DEM	1.48	0.03	0.0384	−0.052	1.10	0.88	0.0252	−0.089	3.30
41	CONGO REP	2.39	0.92	0.0294	−0.152	1.43	0.39	0.0064	−0.118	2.14
42	COSTA RICA	1.51	0.50	0.0142	−0.110	1.08	1.26	−0.0022	−0.209	7.56
43	COTE D’VOIRE	1.47	1.18	0.0309	−0.080	1.35	2.09	0.0253	−0.078	4.19
44	CROTIA	3.95	0.75	−0.0042	−0.069	0.72	0.57	−0.0115	−0.106	6.83
45	CUBA	2.23	0.48	0.0706	−0.063	1.30	0.78	0.0517	−0.040	11.19
46	CURACAO	-	0.50	-	-	-	4.19	−0.0060	−0.074	-
47	CYPRUS	2.21	0.69	−0.0056	−0.131	1.30	0.45	0.0273	−0.089	6.77
48	CZECH	2.40	0.16	0.2570	−0.067	1.22	0.88	0.0474	−0.197	7.65
49	DENMARK	1.60	0.80	−0.0024	−0.087	0.90	0.64	0.0092	−0.048	10.07
50	DJIBOUTI	2.73	0.17	0.0144	−0.094	1.47	0.36	−0.0045	−0.169	2.32
51	DOMINICAN	2.09	1.02	0.0309	−0.088	1.10	1.57	0.0151	−0.081	5.73
52	DOMINICA	-	7.75	-	-	-	0.67	-	-	6.59
53	ECUADOR	2.22	1.46	0.0157	−0.140	1.18	1.14	−0.0045	−0.175	8.14
54	EGYPT	1.69	0.84	0.0527	−0.042	1.33	0.51	0.0243	−0.023	4.95
55	EL SALVADOR	1.58	1.70	0.0783	−0.052	1.29	0.66	0.0535	−0.113	7.11
56	EQUATORIAL G.	10.0	0.38	0.0454	−0.190	2.41	1.48	0.0142	−0.177	3.00
57	ERITREA	2.57	1.18	0.0083	−0.216	0.74	0.80	0.0222	−0.146	4.09
58	ESTONIA	2.10	0.87	−0.0254	−0.116	1.03	3.04	0.0279	−0.099	6.69
59	ESWATINI	2.08	0.94	0.0317	−0.071	1.34	0.71	0.0412	−0.034	6.54
60	ETHIOPIA	2.42	0.80	0.1259	−0.054	1.11	1.24	−0.0041	−0.136	3.30
61	FIJI	-	2.00	-		-	0.50	-	-	3.42
62	FINLAND	1.66	1.14	−0.0030	−0.093	1.04	2.41	−0.0010	−0.119	9.04
63	FRANCE	2.68	1.17	0.2898	−0.110	1.00	2.17	−0.0096	−0.081	11.26
64	GABON	1.83	0.97	0.0404	−0.077	1.44	0.19	0.0187	−0.143	2.75
65	GAMBIA	3.21	0.83	−0.0026	−0.094	2.29	0.37	0.0145	−0.099	3.09
66	GEORGIA	2.19	1.23	0.2536	−0.136	0.76	0.79	0.0293	−0.057	7.11
67	GERMANY	2.84	0.73	0.2624	−0.050	0.98	0.79	0.0050	−0.195	11.43
68	GHANA	1.85	1.48	0.0463	−0.099	1.09	0.62	0.0118	−0.117	3.45
69	GREECE	1.72	0.71	−0.0189	−0.091	1.05	0.71	−0.0111	−0.069	7.72
70	GRENADA	5.78	14.00	-	-	1.08	0.10	0.0106	−0.167	4.46
71	GUADELOUPE	-	1.35	−0.0131	−0.130	-	1.35	−0.0084	−0.137	-
72	GUATEMALA	1.67	0.25	0.0880	−0.044	1.08	0.27	0.1109	−0.197	5.71
73	GUIANA FRENCH	-	0.88	0.0391	−0.102	-	0.43	0.0238	−0.124	-
74	GUINEA	1.50	0.46	0.0097	−0.111	1.36	1.68	−0.0108	−0.126	3.93
75	GUINEA BISSAU	3.56	1.14	0.0230	−0.145	4.66	4.20	-	-	7.00
76	GUYANA	2.49	2.38	0.0005	−0.152	1.54	4.23	−0.0021	−0.163	5.94
77	HAITI	2.32	0.60	0.0565	−0.047	1.66	0.61	0.0217	−0.082	7.69
78	HONDURAS	1.96	0.57	0.0532	−0.086	1.59	1.64	0.0016	−0.141	7.05
79	HONGKONG	-	0.04	−0.0003	−0.060	-	0.24	0.0285	−0.041	-
80	HUNGARY	2.25	0.90	0.0018	−0.093	0.77	1.93	−0.0081	−0.088	6.70
81	ICELAND	2.89	2.28	−0.0261	−0.056	1.86	0.66	−0.0174	−0.079	8.47
82	INDIA	2.43	0.98	0.0331	−0.050	0.91	0.96	−0.0151	−0.048	3.54
83	INDONESIA	2.04	0.95	0.0391	−0.071	1.07	0.99	0.0127	−0.051	2.87
84	IRAN	3.61	1.04	0.2641	−0.063	1.00	0.90	0.0438	−0.140	8.66
85	IRAQ	1.81	0.77	0.1184	−0.084	0.96	0.96	0.0410	−0.150	-
86	IRELAND	2.63	2.16	−0.0021	−0.058	1.45	1.12	0.0188	−0.057	6.93
87	ISRAEL	2.86	0.21	−0.0047	−0.049	1.33	1.16	0.0339	−0.037	7.52
88	ITALY	2.99	1.04	0.2475	−0.040	1.06	3.69	−0.0057	−0.072	8.67
89	JAMAICA	2.43	0.43	−0.0031	−0.089	1.22	2.47	0.0034	−0.174	6.06
90	JAPAN	1.91	1.02	0.0872	−0.055	1.21	1.16	0.0260	−0.052	10.95
91	JORDAN	2.16	2.53	−0.0006	−0.155	0.93	0.93	−0.0138	−0.053	7.79
92	KAZAKHSTAN	2.85	0.60	0.0856	−0.064	1.05	2.06	0.0933	−0.210	2.92
93	KENYA	1.57	1.14	0.0413	−0.067	1.26	1.18	−0.0237	−0.310	5.17
94	KOREA REP.	6.06	1.00	0.1664	−0.076	0.90	1.04	0.0585	−0.090	7.56
95	KOSOVO	1.90	1.02	-	-	0.82	0.99	-	-	-
96	KUWAIT	2.25	0.88	0.0687	−0.031	1.27	1.10	−0.0094	−0.038	5.00
97	KYRGYZSTAN	2.27	0.17	0.0671	−0.091	0.86	1.05	0.0271	−0.200	6.53
98	LAO PDR	-	0.50	-	-	-	0.15	-	-	2.25
99	LATVIA	2.32	0.74	−0.0179	−0.087	1.10	0.50	0.0224	−0.136	6.19
100	LEBANON	1.91	1.03	0.2286	−0.112	1.27	0.90	0.0757	−0.180	8.35
101	LESOTHO	1.99	7.08	0.0053	−0.206	1.36	1.42	0.0398	−0.087	9.28
102	LIBERIA	1.76	0.31	0.0151	−0.114	3.08	4.56	0.0046	−0.159	6.74
103	LIBYA	3.12	0.96	0.0493	−0.047	1.09	0.79	−0.0059	−0.099	-
104	LITHUANIA	1.63	0.83	0.0394	−0.096	0.98	2.49	0.0554	−0.230	6.57
105	LUXEMBOURG	1.99	0.24	−0.0401	−0.061	0.83	1.48	−0.0174	−0.105	5.29
106	MACAO	-	0.29	−0.0019	−0.190	-		-	-	-
107	MADAGASCAR	2.48	0.94	0.0377	−0.057	1.54	0.75	0.0060	−0.211	4.79
108	MALAWI	3.55	1.12	0.0478	−0.088	1.66	6.46	0.0583	−0.087	9.33
109	MALAYSIA	2.86	1.25	0.1042	−0.101	1.15	1.30	0.0794	−0.260	3.76
110	MALDIVES	1.96	0.83	0.0031	−0.154	1.41	1.05	0.0007	−0.116	9.41
111	MALI	1.61	0.64	0.0158	−0.100	0.97	7.78	0.0148	−0.115	-
112	MALTA	4.46	1.06	0.0712	−0.114	1.29	0.99	0.0536	−0.330	8.96
113	MAURITANIA	1.66	1.76	−0.0033	−0.055	0.82	1.14	0.0362	−0.037	4.58
114	MAURITIUS	5.40	4.49	−0.0209	−0.120	9.32	0.35	−0.0032	−0.143	5.83
115	MAYOTTE	-	5.46	0.0129	−0.103	-	1.05	0.0065	−0.154	-
116	MEXICO	2.03	0.86	0.1759	−0.100	0.98	2.53	0.0117	−0.109	5.37
117	MOLDOVA	2.03	1.03	0.0324	−0.086	0.83	0.36	−0.0037	−0.127	6.60
118	MONACO	5.48	3.15	−0.0044	−0.147	1.66	0.54	0.0134	−0.136	1.60
119	MONGOLIA	3.12	10.25	0.0116	−0.204	1.98	0.68	0.0195	−0.127	3.79
120	MONTENEGRO	8.16	1.37	−0.0114	−0.171	1.07	0.66	0.0040	−0.085	8.42
121	MOROCCO	2.05	0.90	0.1161	−0.114	0.84	0.95	−0.0159	−0.065	5.31
122	MOZAMBIQUE	2.14	0.72	0.0260	−0.109	1.59	0.70	0.0152	−0.068	8.17
123	MYANMAR	2.70	1.12	−0.0028	−0.113	0.83	1.15	−0.0137	−0.050	4.79
124	NAMIBIA	2.10	0.68	0.0315	−0.049	1.03	1.22	0.0315	−0.039	7.95
125	NEPAL	2.28	0.74	0.2070	−0.035	0.91	0.78	−0.0264	−0.065	5.84
126	NETHERLAND	2.40	1.19	0.2485	−0.043	0.92	1.04	0.0002	−0.074	9.97
127	NEW CALEDONIA	-	5.00	-	-	-	1.00	-	-	-
128	NEW ZEALAND	5.63	0.74	−0.0426	−0.087	1.89	0.72	0.0140	−0.099	9.21
129	NICARAGUA	5.76	0.97	-	-	1.39	1.02	-	-	8.56
130	NIGER	2.58	0.63	−0.0231	−0.083	0.96	2.21	0.0390	−0.048	7.33
131	NIGERIA	1.91	1.13	0.0502	−0.046	1.06	1.02	0.0333	−0.047	3.89
132	MACEDONIA	1.84	0.74	0.0858	−0.092	0.87	0.74	0.0528	−0.230	6.58
133	NORWAY	2.40	0.77	0.2716	−0.055	1.14	2.13	0.0052	−0.145	10.05
134	OMAN	1.73	3.70	0.0972	−0.092	1.13	9.80	0.0936	−0.130	4.13
135	PAKISTAN	1.90	1.22	0.1301	−0.060	1.02	1.19	0.0113	−0.047	3.20
136	PALESTINE	-	0.96	−0.0053	−0.202	-	1.06	0.0063	−0.050	-
137	PANAMA	2.08	0.96	0.1443	−0.063	1.13	0.79	0.1195	−0.070	7.27
138	PAPAU NEW G.	1.95	0.49	−0.0081	−0.115	2.45	0.88	-	-	2.37
139	PARAGUAY	2.22	0.59	0.0196	−0.147	0.97	1.20	0.0032	−0.168	6.65
140	PERU	2.35	0.89	0.0915	−0.010	1.26	0.53	−0.0077	−0.111	5.24
141	PHILLIPPINES	2.29	1.15	0.1627	−0.082	0.91	1.54	0.1772	−0.174	4.40
142	POLAND	2.17	0.92	0.1562	−0.079	0.99	1.31	0.0094	−0.072	6.33
143	POLYNESIA	-	0.66	-	-	-	0.21	-	−0.075	-
144	PORTUGAL	2.92	1.56	0.0301	−0.140	1.15	3.89	0.0431	−0.190	9.41
145	QATAR	2.61	0.80	0.0694	−0.070	1.16	1.03	−0.0019	−0.094	2.49
146	ROMANIA	2.26	0.88	0.0218	−0.056	0.91	0.95	−0.0072	−0.121	5.56
147	RUSSIA	2.41	1.07	0.0775	−0.020	1.00	0.87	0.0046	−0.037	5.32
148	RWANDA	2.03	1.80	0.0615	−0.146	1.26	0.14	0.0382	−0.064	7.54
149	SAO TOME	3.09	1.44	−0.0218	−0.153	3.33	2.67	0.0162	−0.127	6.27
150	SAN MARINO	5.88	5.10	−0.0157	−0.137	1.14	0.26	−0.0028	−0.154	7.14
151	SAUDI ARABIA	2.31	0.90	0.0607	−0.060	0.90	0.98	−0.0138	−0.029	6.36
152	SENEGAL	2.02	0.72	0.0351	−0.047	1.24	1.59	0.0387	−0.047	3.98
153	SERBIA	2.13	1.62	0.0042	−0.053	0.79	0.82	0.0123	−0.038	8.54
154	SEYCHELLES	2.68	0.48	-	-	1.94	0.54	0.0313	−0.134	5.11
155	SIERRA LEONE	1.50	2.23	0.0143	−0.107	1.52	1.37	0.0291	−0.063	16.06
156	SINGAPORE	2.06	1.33	0.0551	−0.030	1.52	2.83	0.0641	−0.080	4.46
157	SLOVAK	1.74	0.99	−0.0286	−0.123	0.92	0.74	0.0028	−0.193	6.69
158	SLOVENIA	1.78	0.75	−0.0345	−0.079	1.08	0.64	−0.0004	−0.263	8.30
159	SOLOMON ISL.	-	-	-	-	-	0.29	-	-	4.47
160	SOMALIA	1.95	1.18	−0.0085	−0.091	2.55	1.49	-	-	-
161	SOUTH AFRICA	2.54	0.87	0.257	−0.110	1.15	1.72	0.0303	−0.039	8.25
162	SOUTH SUDAN	2.99	0.58	0.0007	−0.152	1.59	0.51	0.0095	−0.133	6.40
163	SPAIN	3.85	0.38	0.3350	−0.035	1.16	0.79	0.0029	−0.080	8.98
164	SRI LANKA	4.14	2.13	0.0144	−0.159	1.04	1.07	0.1347	−0.160	3.76
165	ST KITTS NEVIS	-	2.00	-	-	-	1.00	-	-	5.31
166	ST LUCIA	1.34	1.13	-	-	2.86	0.69	0.0157	−0.082	4.40
167	ST VINCENT	5.86	0.04	-	-	2.17	2.00	0.0407	−0.080	4.47
168	SUDAN	1.97	0.36	0.0193	−0.094	0.24	1.63	0.0407	−0.039	4.51
169	SURINAME	1.41	10.34	0.0214	−0.061	1.27	1.21	0.0379	−0.042	7.97
170	SWEDEN	2.10	0.56	0.2572	−0.106	1.05	0.28	0.0123	−0.162	10.90
171	SWITZERLAND	2.86	1.21	0.2388	−0.044	0.95	0.18	−0.0082	−0.041	11.88
172	SYRIA	2.80	1.43	0.0311	−0.030	1.06	0.66	0.0086	−0.041	-
173	TAIWAN	3.42	1.88	0.0036	−0.084	1.84	1.49	0.0053	−0.123	-
174	TAJIKISTAN	1.68	1.02	0.0418	−0.066	0.54	1.89	−0.0016	−0.131	7.24
175	TANZANIA	5.00	0.91	0.1205	−0.125	18.4	-	-	-	3.63
176	THAILAND	3.42	0.69	−0.0201	−0.055	1.63	2.71	0.0496	−0.100	3.79
177	TIMOR LESTE	-	5.00	-	-	-	1.33	-	-	4.33
178	TOGO	2.09	0.08	0.0093	−0.084	1.41	1.14	0.0083	−0.112	6.17
179	TRINIDAD	5.35	0.32	−0.0025	−0.139	1.30	0.55	−0.0102	−0.113	6.93
180	TUNISIA	2.64	1.53	−0.0122	−0.084	1.15	2.77	0.0053	−0.117	7.29
181	TURKEY	4.32	1.15	0.0120	−0.040	0.81	2.21	0.0078	−0.030	4.12
182	UAE	2.33	0.97	0.0484	−0.080	1.22	1.15	0.0085	−0.055	4.23
183	UGANDA	2.18	0.95	-	-	0.88	0.64	0.0047	−0.154	6.53
184	UKRAINE	2.16	0.96	0.0325	−0.130	0.89	0.30	−0.0032	−0.093	7.72
185	UK	2.89	0.76	0.2223	−0.037	1.25	1.03	0.0106	−0.035	10.00
186	USA	3.85	8.42	0.2882	−0.030	0.99	0.49	0.0121	−0.060	16.89
187	URUGUAY	2.76	0.63	−0.0228	−0.086	1.15	1.03	0.0389	−0.039	9.20
188	UZBEKISTAN	1.82	0.95	0.1231	−0.088	0.71	0.90	0.0238	−0.170	5.29
189	VENEZUELA	2.57	1.54	0.0389	−0.073	0.94	0.82	0.0002	−0.134	3.56
190	VIETNAM	3.59	3.29	−0.0166	−0.158	1.94	1.43	−0.0040	−0.158	5.92
191	VIRGIN ISLANDS	-	0.51	-	-	-	0.33	-	-	-
192	WEST GAZA	3.73	1.00	-	-	0.87	0.98	-	-	-
193	YEMEN	1.57	0.70	0.0049	−0.164	2.84	1.50	0.0006	−0.150	-
194	ZAMBIA	2.80	0.75	0.0265	−0.134	1.73	1.12	0.0372	−0.046	4.93
195	ZIMBABWE	1.98	1.44	0.0367	−0.087	1.40	1.62	0.0438	−0.045	4.73
